# Practices and resilience of dieticians during the COVID-19 pandemic: a national survey in the United Arab Emirates

**DOI:** 10.1186/s12960-021-00682-0

**Published:** 2021-11-20

**Authors:** Farah Naja, Hadia Radwan, Leila Cheikh Ismail, Mona Hashim, Wafaa Helmi Rida, Salma Abu Qiyas, Karen Bou-Karroum, Mohamad Alameddine

**Affiliations:** 1 Department of Clinical Nutrition and Dietetics, College of Health Sciences, Research Institute of Medical & Health Sciences (RIMHS), University of Sharjah, Sharjah, United Arab Emirates; 2 Department of Nutrition and Food Sciences, Faculty of Agricultural and Food Sciences, American University of Beirut, Beirut, Lebanon; 3 Public Health and Prevention Department, Dubai Health Authority, Dubai, UAE; 4 Department of Health Management and Policy, Faculty of Health Sciences, American University of Beirut, Beirut, Lebanon; 5 College of Health Sciences, University of Sharjah, Sharjah, United Arab Emirates

**Keywords:** Dieticians, COVID-19, Resilience, Retention, Satisfaction

## Abstract

**Background:**

The COVID-19 pandemic impacted the practices and resilience of most healthcare workers, including dieticians. In addition to offering critical care to COVID-19 patients, dieticians play a major role in preventing and managing conditions known to affect COVID-19, such as obesity and metabolic disorders. The objective of this study was to examine the conditions and changes in the work environment as well as resilience and its correlates among dieticians during the COVID-19 pandemic in the United Arab Emirates (UAE).

**Methods:**

A cross-sectional national survey was conducted among dieticians practicing in the UAE (*n* = 371), using a web-based questionnaire. The questionnaire addressed, in addition to the sociodemographic information, the practice-related characteristics and resilience of participants. For the latter the Connor–Davidson Resilience Scale© was used. Descriptive statistics as well as simple and multiple linear regressions were used in the statistical analysis.

**Results:**

Of participants, 26.4% reported not having access to personal protective equipment and 50% indicated being concerned for their safety and health. Furthermore, considerable proportions of participants were not satisfied (45%) with the support nor with the appreciation (37.7%) they received during the pandemic. One in four dieticians considered quitting his/her job. While 65.8% of participants reported counseling COVID-19 patients, a third did not use any online platform for counseling. The most cited challenge to dietetic practice during the pandemic was ‘maintaining work-life balance’ (43.1%). The mean CD-RISC score was 72.0 ± 14.0. After adjustment, working in a hospital or public clinic (as opposed to private clinic), having a condition preventing face-to-face counseling, considering quitting job and feeling neutral or dissatisfied with the appreciation were associated with lower resilience scores, while counseling COVID-19 patients was associated with higher scores.

**Conclusions:**

Despite the fairly high resilience among dieticians practicing in the UAE during the COVID-19 pandemic, the findings of this study highlighted a few challenges, mainly related to safe practice environment, support for online counseling, and maintaining work-life balance. Concerted efforts of policy and decision makers ought to develop targeted programs for dieticians to ensure their retention and wellbeing during the COVID-19 pandemic.

## Background

The emergence of the Coronavirus disease 2019 (COVID-19) pandemic has imposed economic, mental, social, and health challenges worldwide. Healthcare professionals have stepped out of their comfort zones and acted on different fronts to help combat the spread of the virus. They have faced excessive stress levels due to the heavy workload, long working hours, work-life imbalance, threat of exposure to the virus, and limited resources [1]. All of these have been recognized as main factors impacting resilience and contributing to increased occupational burnout, depression, anxiety, and emotional exhaustion among healthcare professionals [1, 2].

Dieticians are qualified clinical nutrition professionals who apply the science of food and nutrition with individuals, groups, and communities to support their nutritional needs and prevent/treat their disease conditions [3, 4]. They are integral members of the healthcare team, and they play a role in maintaining the health and wellness of individuals and communities, especially in the care and control of patients with chronic diseases. Similar to other healthcare professionals, dieticians faced personal and professional challenges during the pandemic and were at risk of occupational stress and burnout [2]. Dieticians have played an integral role in caring for COVID-19 patients, especially those who are hospitalized and are at increased risk of malnutrition or wasting during the hospital stay [5]. Dieticians have a role in ensuring the safe delivery of nutrient-rich foods through feeding tubes to help them recover and regain the muscle mass and weight that may have been lost [4]. They are experts in determining the nutritional demands of each patient, taking into account their age, gender, and underlying medical conditions. In addition to that, patients with pre-existing health conditions such as diabetes, cancer, and obesity, also require continuous support from dieticians. As such, dieticians have used telehealth techniques such as video conferencing and online consultations to support these patients during lockdowns [4]. Likewise, dieticians have helped clients in reducing stress through lifestyle, exercise, food, and sleep information and resources; both in-person and virtually to reach the greatest number of clients. Studies reported a 41% increase in the use of telehealth among dieticians for providing care as compared to the period prior to the pandemic [6]. The expectation was that dieticians will adapt rapidly to the changes occurring in the delivery of care; however, several concerns were raised by them, including but not limited to billing and reimbursement, best practices, and health insurance coverage [6].

Despite working directly with people infected with COVID-19, dieticians’ risks are often unrecognized and underestimated [7]. Literature suggests that this lack of support and recognition might negatively affect the well-being of dieticians and their performance [7]. Additionally, job insecurity and uncertainty about the virus might impact dieticians’ quality of life [8]. Recent studies from Brazil revealed that dieticians’ well-being and quality of life have decreased during the pandemic due to poor compensation and lack of professional recognition [7, 8]. The physical and mental well-being of dieticians, similar to other healthcare professionals, is directly linked to their work performance and quality of life. On the same note, healthcare professionals who are experiencing job stressors and low job satisfaction are more likely to quit their jobs and provide poor quality services [9].

Furthermore, dieticians may experience work–family conflict as they attend to both work and family responsibilities. Work–family conflict is defined as a form of inter-role conflict that occurs when the pressure of work demands interferes with personal and family responsibilities [10]. Whereas family–work conflict is a form of inter-role conflict that occurs when the pressure of personal and family life interferes with work responsibilities [10]. A recent study showed that 47.2% of registered dieticians experience work–family conflict and 14.8% experience family–work conflict [3]. It is evident that the presence of work–family conflict is associated with lower life satisfaction, increased burnout, and higher turnover intention. The fact that the dietician workforce is a female majority presents additional pressures and risks since it has been demonstrated in literature that professional working females are disproportionally affected by work-life imbalances and may be at higher risks of professional burnout and turnover [11].

Mitigating the risks of dieticians’ dissatisfaction and turnover, and supporting their work-life balance, should thus be prioritized by the relevant stakeholders. The resilience of healthcare professionals has been gaining prominence in literature, particularly during the COVID-19 outbreak [12, 13]. Resilience, defined as the ability to positively adapt to hardships and significant sources of stress [14], has been recognized as a protective factor against burnout in healthcare professionals [15]. Thus, building resilience has been proposed as an effective approach to improve the well-being of employees [16]. Although much research has been conducted on resilience among physicians, nurses, and other allied healthcare professionals, it has rarely been investigated among dieticians, especially in the Middle East Region.

The United Arab Emirates (UAE) has experienced a rapid transition from a traditional semi-urbanized life to a modern and urbanized one over the past few decades [17]. Along with this transition, the prevalence of obesity and other cardio-metabolic disorders has increased [18, 19]. Dietary change is one of the key strategies to treat and reduce obesity [20]. Dieticians are the key players in this strategy as they are specialized with weight management, in addition to their significant role in treating COVID-19 patients. Therefore, enhancing the resilience of dieticians is essential to improve the health and well-being of the population. The main objectives of this national study were to investigate the conditions and changes of the work environment of UAE dieticians during the COVID-19 pandemic and examine their resilience levels and the factors associated with it. The study aims at suggesting a number of evidence-based policy and practice recommendations that could support the practice of dieticians, enhance their resilience and eventually improve their satisfaction and retention in the UAE labor market.

## Methods

### Study design and subjects’ recruitment

In order to examine the practices and resilience of dieticians in the UAE during the COVID-19 pandemic, a cross-sectional national survey was conducted among dieticians practicing in the country, using a web-based questionnaire. The link for this questionnaire was sent to dieticians by phone and by email. In the absence of an inclusive registry for all dieticians practicing in the UAE, a multitude of sources were sought to maximize the reach of the survey. More specifically, lists of names and contacts of dieticians were obtained from the Ministry of Health (MOH), Dubai Health Authority (DHA), as well as alumni of the various nutrition and dietetics programs in the country. Given that some of these lists may overlap and to avoid duplication of data collection, dieticians were asked not to complete the questionnaire should they have previously participated in this study. Prior to filling the questionnaire, participants read and signed an informed consent which included information about the purpose of the study, its protocol, and time needed to complete the questionnaire. The anonymity and voluntary nature of participation were also stated in the consent form. Once dieticians agreed to participate, they were given the choice to complete the questionnaire in either the English or Arabic language. The study protocol as well as the English and Arabic versions of the consent form and questionnaires were reviewed and approved by the Research and Ethics Committee at the University. Data collection took place between January and May of year 2020.

To be included in the study, a dietician ought to be (1) practicing in the UAE at the time of the survey, and (2) conversant in either the Arabic or English languages. Sample size calculations showed that a total of 385 subjects were needed to estimate a proportion of 50% at 95% confidence and an error margin of 5%. The 50% proportion was selected as a conservative estimate that yields the maximum sample size given that there is no existing information about the prevalence of resilience among dieticians. The formula used in the calculation is as follows:

$$N = {{Z_{\alpha /2}^{2} *p*\left( {1 - p} \right)} \mathord{\left/ {\vphantom {{Z_{\alpha /2}^{2} *p*\left( {1 - p} \right)} {E^{2} }}} \right. \kern-\nulldelimiterspace} {E^{2} }}$$

*N* is the sample size, *Z*_*α*/2_ is the value of the normal distribution that corresponds to a confidence level of 95% where *α* is 0.05 and *Z*_*α*/2_ is 1.96; *p* is the probability of the outcome, which in the case of this study was estimated at 50%; *E* is the margin of error (5%).

### Questionnaire used in data collection

For the data collection, a multi-component questionnaire was used. The latter was developed by a panel of experts consisting of a dietician (practicing in the UAE during the time of the study), a nutrition epidemiologist, a health human resources expert, and a public health nutritionist. The questionnaire was first written in English, later translated to Arabic, and back translated to English. The original and back translated English versions were compared to ensure parallel form reliability.

The first section of the questionnaire included questions related to the sociodemographic characteristics of participants, such as age, gender, marital status, highest level of education attained, years of experience, from which accrediting entity in the UAE the licensure was obtained, having (or not) international licensure to practice, place of practice and the emirate where the dietician worked.

The second section of the questionnaire referred to practice-related characteristics of the participants during the pandemic. A few questions in this section were formulated with a yes/no answer. These questions included: feeling equipped with the required knowledge and skills to practice during the pandemic, having access to personal protective equipment (PPE) and supplies, being concerned about their own safety while on the job, having considered quitting one’s job during the pandemic, and counseling COVID-19 patients. Questions in this section which addressed job satisfaction and feeling appreciated as a practicing dietician during the pandemic were formulated with a 5-point Likert scale answers: very satisfied, satisfied, neutral, dissatisfied and very dissatisfied. Within this section, participants were also asked to rate the efficiency of their practice during the pandemic as compared to before (less efficient, same, more efficient).

The third section of the questionnaire addressed a few reflections on the dieticians’ practices during COVID-19. In this section, information was collected about whether or not participants were engaged in online counseling, the types of online platforms used, and the methods of collecting anthropometric measurement. Participants were also asked to indicate what skills they needed to improve in online counseling, challenges to work as a dietician during the pandemic, and changes in patients’ numbers and types.

The last section of the questionnaire addressed the resilience of study participants, which was examined using the Arabic version of the Connor–Davidson Resilience Scale© (CD-RISC). The latter consisted of 25 items, each rated on a 5 point-Likert scale ranging from not true at all to true nearly all the time. These items examined participants’ resilience across five main domains: (1) personal competence, high standards, and tenacity, (2) trust in one’s instinct, tolerance of negative effects, and strengthening effects of stress, (3) positive acceptance of change and secure relationships, (4) Control and (5) Spiritual influences. The response for each of the items were assigned points as follows: Not true at all (0 point), rarely true (1 point), sometimes true (2 points), often true (3 points), true nearly all the time (4 points). For each participant, a total score for resilience was computed as the sum of points obtained on each of the items. The total score ranged between 0 and 100, with higher scores indicating better resilience [21, 22].

The developed questionnaire (both English and Arabic versions) was pilot tested on a sample of 15 dieticians who were practicing at the time of data collection. The pilot testing served to ensure the face validity of the questionnaire as well as the cultural acceptability of its content. The results of the pilot test phase were not included in the analysis for this study.

### Statistical analysis

The IBM SPSS statistics software version 25 for Windows was used to carry out data analysis. Categorical data were depicted as frequencies and percentages, while means ± standard deviations were used to describe numerical data. Simple and multiple linear regression analyses were conducted to examine the correlates of resilience in the study population. For the regression analyses, the resilience score was the dependent variable while sociodemographic and practice related characteristics were the independent variables. In addition to age and gender, variables that were significantly associated with resilience in the simple regression analyses were entered in the multiple regression model. Results of the linear regression analyses were expressed as regression coefficient (*β*) with 95% Confidence Intervals (CI). *p*-values less than 0.05 were considered statistically significant.

## Results

A total of 371 questionnaires were completed and returned electronically, of which data was extracted and used in the analysis. Table 1 described the characteristics of the study participants. The sample had a higher proportion of female participants as compared to males (87.6% vs. 12.4%) and consisted of rather young dieticians, with participants 45 years and older making up only 14.4% of the total sample. A bit shy of two thirds of respondent had a Bachelor of Science degree (65.2%). Around 40% of participants reported being licensed by the MOH, 32% by DHA, while 18.1% of participants had a license to practice from another country in addition to that from the UAE. More than half of responding dieticians worked in hospitals (56.1%) while the rest were distributed almost equally between private and public health clinics (Table 1).

**Table 1:** Demographic and professional characteristics of the study population (*n* = 371)

Characteristics	Frequency	Percent
Gender		
Female	325	87.6
Male	46	12.4
Age (years)		
19–29	123	33.4
30–44	192	52.2
≥ 45	53	14.4
Marital status		
Ever married	230	62.0
Never married	141	38.0
Do you have children?		
No	171	46.1
Yes	200	53.9
Number of children		
1	44	22.4
2	77	39.3
3	27	13.8
4	22	11.2
≥ 5	26	13.3
Highest education level		
BSc	242	65.2
Masters/PhD	129	34.8
Years of experience		
Less than 5 years	132	38.3
More than 5 years	213	61.7
Where did you obtain your local licensed from?		
Ministry of Health and Prevention	150	40.4
Dubai Health Authority	119	32.1
Health Authority of Abu Dhabi	53	14.3
Dubai Health Care City	9	2.4
From more than one of the above places	40	10.8
Do you have international licensing certificate?		
No	304	81.9
Yes	67	18.1
Where do you practice?		
Hospital-based	208	56.1
Private clinic	65	17.5
Public health clinic	63	17.9
Other	16	4.5
Emirate working in		
Abu Dhabi	85	22.9
Dubai	171	46.1
Other emirates	115	31.0

The practice-related characteristics during the COVID-19 pandemic of study respondents are described in Table 2. Almost 14% of participants indicated that they do not feel well equipped with the required knowledge and skills to practice during a pandemic and 26.4% reported not having access to PPE and supplies to safely carry out the work with patients. Half of the responding dieticians reported being concerned to a large extent for their safety and health while practicing during the pandemic. Furthermore, 45% of respondents were either neutral or dissatisfied with the support they received for practicing during the pandemic. In addition, 37.7% of respondents also reported being neutral or dissatisfied with the appreciation they received during the pandemic. Among participants, 1 in 4 dieticians had considered quitting his/her job. While 68.7% of respondents indicated working from home, 65.8% reported counseling COVID-19 patients (Table 2).

**Table 2:** Practice-related characteristics during the COVID-19 pandemic among study participants (*n* = 371)

	Frequency	Percent
As a dietician, do you feel well equipped with the required knowledge and skills to practice during a pandemic?		
No	51	13.7
Yes	320	86.3
Do you have adequate access to personal protective equipment and supplies to safely carry out your work with patients?		
No	98	26.4
Yes	273	73.6
Are you concerned about your health and safety while practicing as a dietician during the COVID-19 pandemic?		
Yes, to a larger extent	191	51.5
Yes, to a lesser extent/no, I am not concerned at all	180	48.5
Do you have any condition that prevents you from face-to-face counseling?		
No condition	270	72.8
Yes^a^	101	27.2
How satisfied are you with the support received for your practice as a dietician during the COVID-19 pandemic?		
Very satisfied/satisfied	204	55.0
Neural/dissatisfied/very dissatisfied	167	45.0
During the COVID-19 pandemic, have you considered quitting your job?		
No	280	75.5
Yes	91	24.5
Since the start of the COVID-19 pandemic, which of the following statements describes your situation?		
I am mainly working from home/blended	116	31.3
I am mainly working at my institution/office/no change	255	68.7
Rate the efficiency of dietetic counseling after COVID-19 pandemic as compared to before COVID-19 pandemic		
Same/less efficient	236	63.6
More efficient	135	36.4
How appreciated do you feel as a practicing dietician during the COVID-19 pandemic?		
Very satisfied/satisfied	231	62.3
Neutral/dissatisfied/very dissatisfied	140	37.7
Have you counseled COVID-19 patients?		
No	127	34.2
Yes	244	65.8

^a^Pregnancy, chronic disease, elderly in the house

Table 3 outlined the reflections of study participants with regards to practicing during the pandemic. With regards to online counseling, a third of the responding dieticians did not use any online platform for counseling while 61.4% and 36.2% indicated they needed to improve their online counseling techniques and their computer-based skills for online platforms such as Zoom and Microsoft Teams, respectively. Of dieticians who conducted online counseling, 11.8% did not follow up on anthropometric measurements of their patients and 60.2% relied on self-reported measurements by their patients. Among the challenges to dietetic practice during the pandemic, maintaining work-life balance was reported by 43.1% of participants, followed by keeping positive energy (39.6%) and maintaining face to face counseling (39.6%). Half of the dieticians participating in this study (51.5%) indicated a decrease in the number of patients seen during the pandemic as compared to before. As for the type of patients, while 36.7% reported no change, 22.9% and 19.1% indicated seeing more patients with metabolic disorders and weight related problems, respectively.

**Table 3:** Dieticians’ reflections on practices during the COVID-19 pandemic among study participants (*n* = 371)

	Frequency	Percent
Have you used online platforms for counseling your patients in your dietetic practice?		
No	125	33.7
Yes	246	66.3
Type of platforms used (choose all that applies)		
Zoom	191	51.5
MS Teams	98	26.4
WebEx	33	8.9
WhatsApp	113	30.5
Other	19	5.1
How did you follow up on the anthropometric measurements of your patients while doing online counseling?		
I did not follow up on the anthropometric measurements	29	11.8
Patients self-weighing	148	60.2
Using hospital/clinical machines	67	28.0
What skills do you think you need to improve your dietetic practice during COVID-19? (More than one option can be selected)		
Online counseling skills (engaging patients virtually)	262	61.4
Computer-based skills (Zoom, MS Teams, etc.)	157	36.2
Other	15	3.5
Since the start of the COVID-19 pandemic, what are the most challenging aspects for your work as a dietician? (Choose all that are applicable)		
Maintaining work-life balance	160	43.1
Keeping positive energy	147	39.6
Face-to-face counseling	147	39.6
Seeing enough patients	117	31.5
Job security	99	26.7
Job satisfaction	75	20.2
No challenges	45	12.1
Which statement accurately reflects the changes in the number of patients consulting with you during the COVID-19 pandemic?		
The number of patients I am following decreased	191	51.5
The number of patients I am following increased	97	26.1
There is no change in the number of patients I am following	83	22.4
During the COVID-19 pandemic, what type of patients are you seeing more as compared to before COVID-19 pandemic?		
No change in the type of patients	136	36.7
Metabolic (Diabetes, CVD, HTN)	85	22.9
Weight loss	71	19.1
Eating disorders	32	8.6
Renal	22	5.9
Other	25	6.7

**Fig. 1:**
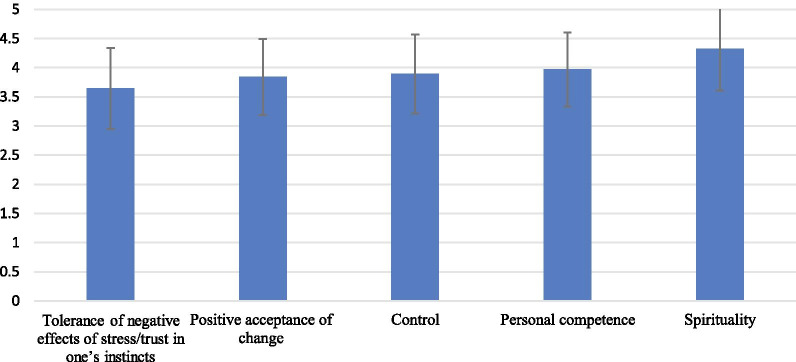
Scores on the five domains of resilience among study participants, as measured by the CD-RISC (mean ± SD).

With regards to the results of the resilience assessment, the mean score of the CD-RISC was 72.0 ± 14.0. As for the internal consistency of this questionnaire, the Cronbach alpha of the 25 items was 0.946. The means ± SD of the scores corresponding to the five resilience domains as measured by the CD-RISC are presented in Fig. 1. Spirituality had the highest mean score (4.3) followed by the personal competence, high standards, and tenacity (3.9). Trust in one’s instinct, tolerance of negative effects, and strengthening effects of stress had the least mean score (3.6).

After adjustment, the results of the multiple linear regression presented in Table 4 showed that, compared to dieticians practicing in private clinics, those working in hospitals or public clinics had significantly lower resilience score (*β* = − 5.4, CI − 9.24, − 1.48 and *β* = − 5.7, CI − 10.53, − 0.92, respectively). In addition, dieticians having a condition preventing them from face-to-face counseling had lower resilience scores (*β* = − 3.7, CI − 7.01, − 0.41). Better resilience was also negatively associated with considering quitting job (*β* = − 5.0, CI − 8.39, − 1.58) and with feeling neutral or dissatisfied with the appreciation received as a practicing dietician during the pandemic (*β* = − 3.2, CI − 6.27, − 0.13). A positive association was observed between counseling COVID-19 patients and resilience (*β* = 4.4, CI 1.23, 7.62).

**Table 4:** Association of RISC scores with the various characteristics in the study population, as examined by simple and multiple linear regressions (*n* = 371)

	*ß* (95% CI)^a^	Adjusted *ß* (95% CI)^a*^
Gender		
Female	–	
Male	− 0.80 (− 5.30, 3.70)	
Age		
19–29 years	–	
30–44 years	0.18 (− 3.13, 3.49)	
≥ 45 years	1.3 (− 3.44, 5.98)	
Marital status		
Ever married	–	
Never married	− 2.0 (− 5.09, 1.0)	
Do you have children?		
No	–	
Yes	− 0.51(− 3.49, 2.46)	
Highest education level		
BSc	–	
Masters/PhD	2.6 (− 0.50, 5.71)	
Years of experience		
Less than 5 years	–	
More than 5 years	− 1.3 (− 4.52, 1.86)	
Having an international license		
No	–	
Yes	0.13 (− 3.73 to 3.98)	
Where do you practice?		
Private clinic	–	–
Hospital-based	**− 4.8 (− 8.80, − 0.80)**	**− 5.4(− 9.24, − 1.48)**
Public health clinic	**− 7.0 (− 11.96, − 2.01)**	**− 5.7 (− 10.53, − 0.92)**
Other	2.8 (− 5.01, 10.70)	5.5 (− 2.11, 13.10)
Emirate working in		
Abu Dhabi	–	
Dubai	1.5 (− 2.26, 5.33)	
Other	1.2 (− 2.90, 5.28)	
Do you have adequate access to personal protective equipment and supplies to safely carry out your work with patients?		
No	–	–
Yes	3.0 (− 0.33,6.37)	
Are you concerned about your health and safety while practicing as a dietician during the COVID-19 pandemic?		
Yes, to a larger extent	–	
Yes, to a lesser extent/No, I am not concerned at all	2.0 (− 1.0, 4.93)	
Do you have any condition that prevents you from face-to-face counseling?		
No	–	
Yes	**− 4.1 (− 7.31, − 0.91)**	**− 3.7 (− 7.01,− 0.41)**
How satisfied are you with the support received for your practice as a dietician during the COVID-19 pandemic?		
Very satisfied/satisfied	–	
Neural/dissatisfied/very dissatisfied	− 2.2 (− 5.13,0.82)	
During the COVID-19 pandemic, have you considered quitting your job?		
No	–	
Yes	**− 4.8 (− 8.21, − 1.38)**	**− 5.0 (− 8.39, − 1.58)**
Since the start of the COVID-19 pandemic, which of the following statements describes your situation?		
Home/blended	–	
Working at my institution/no change	− 2.10 (5.29, 1.09)	
Rate the efficiency of dietetic counseling after COVID-19 pandemic compared to before COVID-19 pandemic		
Same/less efficient	–	
More efficient	**3.6 (0.51, 6.64)**	0.12(− 3.01, 3.26)
How appreciated do you feel as a practicing dietician during the COVID-19 pandemic?		
Very satisfied, satisfied	–	
Neutral/dissatisfied/very dissatisfied	**− 4.0 (− 7.0, − 0.92)**	**− 3.2 (− 6.27, − 0.13)**
Have you counseled COVID-19 patients?		
No	–	
Yes	**4.3 (1.22,7.41)**	**4.4 (1.23,7.62)**

^a^*ß* represents the linear regression coefficients. Significant *ß* (< 0.05) are bolded

^*^Adjusted for age, gender and variables that were significant (0.05) in the simple linear regression were entered in the multiple regression model

## Discussion

To the knowledge of the authors, this is the first manuscript to investigate the practice environment of UAE dieticians and their resilience levels during the COVID-19 pandemic. Study findings showed that half of the dieticians were to a large extent concerned for their safety and health while practicing and a comparable proportion did not feel that their practice is adequately supported during the pandemic. Close to two thirds of dieticians were involved in caring for COVID-19 patients and 38% were either neutral or dissatisfied with the appreciation they received for their services. Perhaps most disconcerting was the fact that a quarter of the surveyed dieticians indicated consideration for quitting their jobs. Three out of each five dieticians expressed the need to enhance their online counselling skills and confirmed the subjective reporting of anthropometric measurements by the patients they were counselling during the pandemic. Among the challenges to dietetic practice during the pandemic, maintaining work-life balance was the top challenge (43.1%) followed by keeping positive energy (39.6%) and maintaining face-to-face counseling (39.6%). Dieticians reported relatively high resilience scores with the spirituality domain being the highest and the trust in one’s instinct, tolerance of negative effects, and strengthening effects of stress being the lowest. While counselling COVID-19 patients was positively associated with higher resilience scores, hospital employment, having a condition preventing face-to-face counselling, considering quitting job and being neutral or dissatisfied with appreciation received during the pandemic were negatively associated with resilience scores.

The findings of this study revealed that dieticians were expecting a higher level of appreciation and support for their practice during the COVID-19 pandemic, especially that most of them cared for COVID-19 patients. While much focus was dedicated to appreciating and supporting frontline physicians and nurses caring for the COVID-19 patients during the pandemic [23], and rightfully so, supporting the practice of dieticians may not have been viewed as a comparable priority despite their valuable services to COVID-19 patients. The lack of support became more evident with 45% of respondents being either neutral or dissatisfied with the support they received for practicing during the pandemic.

Support during the pandemic is not a luxury, especially if it safeguards an optimal delivery of patient care. It is disconcerting that half of the responding dieticians expressed a large degree of concern over the safety of their practice. Safe practice enables at the basic level providing dieticians who maintained face-to-face counselling with adequate access to PPE (26% indicated not having adequate access) and supporting dieticians working from home with training on effective techniques for safe and secure online counselling. Such support extends beyond the basic training on using online conferencing software to valid and effective collection of anthropometric measurements using digital technologies and online tools. Tools that may improve telemedicine services by dieticians include pedometers and wearables, electronic home-scales, and continuous glucose monitoring. On the same note, the adoption of digital health platforms such as the Academy of Nutrition and Dietetics Health Informatics Infrastructure (ANDHII), an electronic web-based tool, can allow dieticians to remotely monitor and evaluate their patients [24].

Study findings also revealed that the level of dissatisfaction during the pandemic has not only affected the delivery of safe and effective patient care, but also caused the destabilization of the workforce with a quarter of surveyed dieticians indicating consideration for quitting their jobs. A comparable proportion of dieticians indicated concerns over their job security. Furthermore, two out of each five dieticians indicated challenges in maintaining work-life balance, maintaining positive energy, and offering face-to-face counselling. The above-mentioned results call on concerned public and private decision and policy makers to immediately and effectively respond to the needs of dieticians to enable them to maintain safe practice, enhance their satisfaction and improve their retention.

Resilience has been proven in previous studies to mitigate the negative effects of the work environment including intentions to quit [25]. Yet such an investigation has not been previously carried out on dieticians in the Middle East region. The mean resilience score in this study was 72.0 ± 14.0, which falls relatively higher as compared to investigations carried out on other health professionals in the region [26, 27]. Zooming into the specific resilience domains, analysis revealed that spirituality had the highest mean score followed by the personal competence, high standards, and tenacity. Spirituality plays an important role in the Middle East region, particularly among Muslim populations [28]. Previous studies have reported significant associations between spirituality and mental health outcomes such as anxiety, depression, self-esteem, hope and optimism, and overall well-being [29]. Further studies from the Middle East also revealed that spirituality encompasses both personal and professional lives of individuals [28]. We could thus infer that spirituality played a significant role in dieticians’ ability to cope with the COVID-19 pandemic. While participants in this study had a relatively high rating in their competence as dieticians, they had the lowest rating for trust in one’s instinct, tolerance of negative effects, and strengthening effects of stress. The findings, hence, highlight an opportunity for policy and decision makers to support the dietician workforce by offering stress relief programs to mitigate the negative effects of the pandemic. These programs have been implemented elsewhere, covering several areas: providing leisure activities, training on psychological skills to deal with multiple stressors, ensuring regular visits from psychological counsellors to support staff and listen to their stories, and providing a safe place where healthcare workers can rest and temporarily isolate themselves from their families when needed [30]. Implementing such psychological support interventions is expected to help dieticians reduce stress to better respond to the pandemic.

In this study, counselling COVID-19 patients was positively associated with higher resilience scores. This finding suggests that providing services to COVID-19 patients decreases fear and enhances the sense of service for care professionals. It has been suggested that individuals who are able to help at times of crisis are usually better able to cope and overcome negative feelings [31]. A study describing the experiences of healthcare professionals during the COVID-19 pandemic revealed that a sense of responsibility to reduce patients’ suffering and safeguard the country was pervasive throughout their stories [32]. This sense of responsibility has been proved to act as a powerful catalyst for building resilience [33].

Analysis of study findings showed that hospital employment, having a condition preventing face-to-face counselling, considering quitting job and being neutral or dissatisfied with appreciation received during the pandemic were negatively associated with resilience scores. Working in a hospital (especially in the absence of adequate access to PPE), and having a chronic condition increase the risk of developing serious and severe symptoms if COVID-19 is contracted and is thus negatively associated with resilience. Customized support programs need to be offered to dieticians with chronic conditions and for those working in hospitals to enhance their resilience. Providing support programs falls under the purview of both the institution and the government. Health institutions have a role in ensuring the availability of essential facilities to ease dieticians’ workload during these difficult times. Such facilities include installing an efficient, user friendly, and secure information technology infrastructure to ensure easy access for relevant databases and information from remote locations [34]. Moreover, institutions should incorporate policies and strategies to promote flexible practices that enable dieticians to juggle between their work and family responsibilities, thereby decreasing the psychological stress and enhancing resilience [34]. Likewise, the government has a role in reducing the pressure on dieticians along with other healthcare professionals, through providing PPE, reducing the workload intensity of dieticians, adopting strict infection control measures, and offering practical guidance [35]. Based on previous studies, these measures helped protect the mental health of healthcare professionals during previous outbreaks [34, 35]. Finally, and as per the results of previous studies, resilience was negatively associated with intention to quit [34, 36] and with absence of adequate support [34]. These findings reinforce the importance of extending adequate and customized support to dieticians during the pandemic. Such support would not only enhance the resilience of dieticians, but would also improve their retention [34].

The results of this study did not show any associations between the demographic characteristics of the participants (age, sex and years of experience) with resilience of dieticians. While this finding may not be in line with previous research, it arguable that during the COVID-19 pandemic, demographic factors such age and sex, have less of an effect on resilience while other factors more directly linked to the pandemic exert a significant influence on resilience. In fact, the results of this study showed significant associations among many COVID-19-related characteristics and the resilience of dieticians, such as ‘presence of any condition that prevents you from face-to-face counseling’, ‘the efficiency of dietetic counseling after COVID-19 pandemic compared to before COVID-19 pandemic’, and ‘Have you counseled COVID-19 patients’.

The findings of this study ought to be considered in light of a few limitations. First, the absence of a national registry of dieticians in the country could have limited the reach of subjects’ recruitment. That said, the sample size as well as the diverse age ranges, educational levels and place of practice of participants supports the representativeness and generalizability of the results. Second, the COVID-19 lockdown and restrictions imposed in the UAE during data collection imposed the online nature of the data collection in this study. Though this method of data collection method eliminates potential interviewer bias and social desirability bias, it is possible that participants may have misread or misunderstood certain questions. Third, our sample is skewed towards younger age. Although, there is no comprehensive registry of dieticians in the UAE in order to compare its age distribution with our sample, the overall population age distribution of the UAE is generally skewed towards a younger age. In 2020, the forecasted largest age group in the UAE was those between 30 and 34 years, at around 1.8 million. The second largest age group in the country was between 25 and 29 years. [37]. Lastly, as this study was conducted using a cross-sectional design, the results could not imply any cause-effect relationship as reverse causality remains a possibility.

## Conclusion

This is the first study to characterize the conditions and changes in the work environment as well as resilience levels of dieticians during the COVID-19 pandemic in the UAE. The findings showed that, despite counseling COVID-19 patients, considerable proportions of dieticians indicated a high level of concern for one’s safety, were not satisfied with the institutional support or were not appreciated during their practice. In line with these findings, the results of this study showed that 25% of dieticians are considering quitting their jobs. Such results were observed despite the fairly good overall resilience levels of the dieticians found in this study, which were mainly driven by a high level of spirituality and personal competence. Improving trust in one’s instinct, tolerance of negative effects as well as maintaining work-life balance were areas of improvement identified in this study. Taken together, the findings of this study call for concerted efforts to ensure safe practice conditions for dieticians during the pandemic, including but not limited to, providing self-protecting equipment and training on digital health to allow remote counseling for patients. In addition, the findings highlight an opportunity for policy and decision makers to support the dieticians’ workforce by offering stress relief programs (such as leisure activities and training on psychological skills to deal with multiple stressors) to mitigate the negative effects of the pandemic.

## Data Availability

The datasets generated and/or analysed during the current study are not publicly available due privacy and ethical restrictions but are available from the corresponding author on reasonable request.
